# Circulating vaccine-derived poliovirus: a menace to the end game of polio eradication

**DOI:** 10.1186/s12992-020-00594-z

**Published:** 2020-07-16

**Authors:** Long Chiau Ming, Zahid Hussain, Siang Fei Yeoh, David Koh, Kah Seng Lee

**Affiliations:** 1 PAP Rashidah Sa’adatul Bolkiah Institute of Health Sciences, Universiti Brunei Darussalam, Jalan Tungku Link, Gadong, BE1410 Brunei Darussalam; 2 Discipline of Pharmacy, School of Health Sciences, Faculty of Health, University of Canberra, Canberra, Australia; 3 Department of Pharmacy, National Hospital Health System, Singapore, Singapore; 4 SSH School of Public Health, National University of Singapore, Singapore, Singapore; 5 Faculty of Pharmacy, University of Cyberjaya, Cyberjaya, Selangor Malaysia

**Keywords:** Immunization, Vaccination, Poliomyelitis, Vaccine supply and distribution, Vaccine control, oral polio vaccine, inactivated polio vaccine

## Abstract

The World Health Organisation Western Pacific Region countries were declared free of polio in 2000 until a polio outbreak involving 305 cases occurred in Indonesia in 2006. It was not until 2014 that the World Health Organisation South East Asia region was officially declared polio-free again. However, in February 2019, the Global Polio Eradication Initiative announced a new circulating vaccine-derived poliovirus outbreak in the Papua province of Indonesia. To make matter worse, the outbreak responses were tardy and led to transmission among migrating communities to other cities. The pressing regional issues of polio outbreak caused by circulating vaccine-derived poliovirus and use of oral polio vaccine have not been well presented. Our letter highlighted the suboptimal outbreak responses as well as the necessity of cross-border vaccination to curb continued poliovirus transmission.

Until recently, there had been no reported cases of polio since 1978 in Brunei [1], since 1992 in Malaysia and since 1993 in the Philippines [2]. The World Health Organization (WHO) Western Pacific Region countries were declared free of polio in 2000 until a polio outbreak involving 305 cases occurred in Indonesia in 2006 [3]. However, in 2019, polio re-emerged in three countries in the Western Pacific region. The Global Polio Eradication Initiative announced a new circulating vaccine-derived poliovirus (cVDPV) outbreak in Papua, Indonesia in Feb 2019. In September 2019, a national polio outbreak was declared in the Philippines [4]. Subsequently, on December 6, 2019, the WHO’s Polio Regional Laboratory in Australia verified that the sample taken from an infant from the Malaysia Borneo state of Sabah was infected with cVDPV which had a direct link to the polio virus detected in the acute flaccid paralysis cases reported in the neighboring Filipino island of Mindanao [4].

## Main cause of re-emergence

cVDPV remains a menace to the end game of polio eradication as it is one of the main causes of re-emerging polio apart from vaccination refusal. cVDPV originates from the oral polio vaccine. This weakened virus is excreted by the body through feces which could spread easily in a non-sanitary environment. In the early 2000s, in order to avoid the risk of cVDPV posed by the weakened poliovirus transmitted through oral polio vaccine (OPV), inactivated polio vaccine (IPV) was strongly advocated. By June 2018, 39 of 47 African countries had changed from OPV to IPV. In spite of that, sporadic cases of cVDPVs were spotted in countries using OPV with low population immunity [5]. For example, in 2018, cVDPV outbreaks were reported in Nigeria, Kenya and the Democratic Republic of the Congo,

Another approach to totally eliminate the risk of reemergence of wild poliovirus type-2, is to utilize bivalent OPV that contains Sabin serotypes 1 and 3 strains, instead of trivalent OPV that contains Sabin strains of all three poliovirus serotypes [6].{Regional Committee for Africa (68), 2018 #6} Drastic measures were taken when all member states in the WHO African Region withdrew trivalent OPV in May 2016 [5]. In Southeast Asia, Brunei and Malaysia has fully utilized the IPV but the trivalent OPV is still used in the Philippines and Indonesia with the latter introducing IPV in the national immunization program from July 2016 [7]. Furthermore, the use of monovalent type-2 polio vaccine in response to cVDPV Type-2 outbreaks poses the risk of the emergence of “de-novo” cVDPV Type-2 [8].

Besides the use of IPV or bivalent OPV, a comprehensive vaccine coverage is essential. Even though in general Brunei and Malaysia had achieved more than 95% immunization rates, approximately 12% of the children living in the affected sites on the coastal region of Sabah were not vaccinated, putting the whole community at risk of losing the vital herd immunity. The situation is heterogenous as across the South China Sea, the immunization rate among 105 million residents in the Philippines stood at 66 and 41% for OPV and IPV, respectively [9]. In the remote Papua province of Indonesia, polio vaccination coverage was 68.2% in 2017 and 40.8% in 2018 [10].

### Cross-border polio vaccinations: the urgent need in southeast Asian countries

Cross border cooperation and efforts are needed. The mapping of less accessible border areas e.g. in Papua New Guinea and Timor Leste as well as migrant routes is important. The vaccination should be given by humanitarian groups to stateless children or non-citizens. It is especially important to maintain coverage of polio immunization at over 95% in high-risk areas with seasonal migrant populations. Surveillance of vaccination patterns based on data mining and geo-mapping of migrant and cross-border vaccinations that has proven successful in containing polio in Nigeria could be adopted to optimize the limited vaccine stocks and healthcare resources.

## Conclusion

The health authorities need to increase the inactivated polio vaccine coverage to prevent further spread of cVDPV. Doctors should always look out for polio-like symptoms of acute flaccid paralysis among children by testing the presence of polio virus or cVDPV in the patients’ stool samples. Other important precautionary measure is to test the presence of polio virus or cVDPV at sewage treatment plants. Application of advanced analytics and disease transmission model to map out and estimate seasonal migrant routes and populations could save time and lives of the vulnerable populations. Despite of inactivated polio vaccine stock-outs and cost of polio vaccination, cross-border vaccination at permanent transit point and surveillance of nomadic populations would still be cost-effective. The existence of inadequately vaccinated groups within neighbouring countries pose a serious threat to reintroduction of the poliovirus.

## Data Availability

All data have been included.

## Abbreviations

OPV: Oral polio vaccine
IPV: Inactivated polio vaccine
cVDPV: Circulating vaccine-derived poliovirus
WHO: World Health Organization
